# RNN and BiLSTM Fusion for Accurate Automatic Epileptic Seizure Diagnosis Using EEG Signals

**DOI:** 10.3390/life12121946

**Published:** 2022-11-22

**Authors:** Nagwan Abdel Samee, Noha F. Mahmoud, Eman A. Aldhahri, Ahsan Rafiq, Mohammed Saleh Ali Muthanna, Ijaz Ahmad

**Affiliations:** 1 Department of Information Technology, College of Computer and Information Sciences, Princess Nourah bint Abdulrahman University, Riyadh 11671, Saudi Arabia; 2 Rehabilitation Sciences Department, Health and Rehabilitation Sciences College, Princess Nourah bint Abdulrahman University, Riyadh 11671, Saudi Arabia; 3 Department of Computer Science and Artificial Intelligence, College of Computer Sciences and Engineering, University of Jeddah, Jeddah 23218, Saudi Arabia; 4 School of Automation, Chongqing University of Posts and Telecommunications, Chongqing 400065, China; 5 Institute of Computer Technologies and Information Security, Southern Federal University, 347922 Taganrog, Russia; 6 CAS Key Laboratory of Human-Machine Intelligence-Synergy Systems, Shenzhen Institute of Advanced Technology, Chinese Academy of Sciences, Shenzhen 518055, China; 7 Shenzhen College of Advanced Technology, University of Chinese Academy of Sciences, Shenzhen 518055, China; 8 Guangdong-Hong Kong-Macao Joint Laboratory of Human-Machine Intelligence-Synergy Systems, Chinese Academy of Sciences, Shenzhen 518055, China

**Keywords:** recurrent neural network, bi-directional long short-term memory, electroencephalographic, epileptic seizure, deep learning, machine learning

## Abstract

Epilepsy is a common neurological condition. The effects of epilepsy are not restricted to seizures alone. They comprise a wide spectrum of problems that might impair and reduce quality of life. Even with medication, 30% of epilepsy patients still have recurring seizures. An epileptic seizure is caused by significant neuronal electrical activity, which affects brain activity. EEG shows these changes as high-amplitude spiky and sluggish waves. Recognizing seizures on an electroencephalogram (EEG) manually by a professional neurologist is a time-consuming and labor-intensive process, hence an efficient automated approach is necessary for the identification of epileptic seizure. One technique to increase the speed and accuracy with which a diagnosis of epileptic seizures could be made is by utilizing computer-aided diagnosis systems that are built on deep neural networks, or DNN. This study introduces a fusion of recurrent neural networks (RNNs) and bi-directional long short-term memories (BiLSTMs) for automatic epileptic seizure identification via EEG signal processing in order to tackle the aforementioned informational challenges. An electroencephalogram’s (EEG) raw data were first normalized after undergoing pre-processing. A RNN model was fed the normalized EEG sequence data and trained to accurately extract features from the data. Afterwards, the features were passed to the BiLSTM layers for processing so that further temporal information could be retrieved. In addition, the proposed RNN-BiLSTM model was tested in an experimental setting using the freely accessible UCI epileptic seizure dataset. Experimental findings of the suggested model have achieved avg values of 98.90%, 98.50%, 98. 20%, and 98.60%, respectively, for accuracy, sensitivity, precision, and specificity. To further verify the new model’s efficacy, it is compared to other models, such as the RNN-LSTM and the RNN-GRU learning models, and is shown to have improved the same metrics by 1.8%, 1.69%, 1.95%, and 2.2% on using 5-fold. Additionally, the proposed method was compared to state-of-the-art approaches and proved to be a more accurate categorization of such techniques.

## 1. Introduction

One of the most frequent long-lasting neurological disorders is epilepsy. The impacts of epilepsy are far-reaching; they are not limited to the physical manifestations of seizures alone. Rather, they encompass a wide range of difficulties that can lead to impairment and a drastically diminished quality of life [1]. Having epilepsy can have a negative impact on your emotional, mental, and physical well-being. People with epilepsy, and especially those with refractory epilepsy (i.e., seizures that are not well-controlled with seizure medications), face a number of challenges, including a lack of social support and family function, difficulties with cognition, medical, and psychiatric co-occurring disorders, and stigma [2,3]. Epilepsy is a neurological disorder disease that affects over 50 million individuals all over the world [4], and unfortunately, the therapies that are now available, such as anticonvulsants and surgery, can have significant negative consequences on the patients who undergo them. These sudden discharges momentarily interrupt both the patient’s behavior and their ability to function normally. Epilepsy cannot be brought under complete control with the therapies, drugs, or surgical procedures that are now available [5,6,7].

Seizures can strike without warning, and epilepsy can have a significant impact on a person’s social and psychological life; as a result, the condition is regarded as potentially fatal [8]. Therefore, epileptic seizure prediction significantly contributes to an increase in the quality of life of seizure patients through a number of different aspects. These aspects include the generation of an alarm to offer the appropriate action prior to the occurrence of a seizure, the introduction of new treatment approaches, and the developing of new strategies for understanding the nature of the disease [9,10]. In general, conventional therapy for epileptic seizures frequently results in a variety of unwanted side effects, which makes it exceptionally challenging to maintain seizure control in patients [11,12]. According to estimates provided by the World Health Organization (WHO), approximately fifty thousand people have epilepsy in the world [13]. In addition, it is said that 70% of all occurrences of epilepsy-related seizures occur in poor nations, which means that the drugs and treatment facilities necessary for accurately diagnosing epilepsy are not always available.

Due to the fact that epilepsy is a disorder associated with the electrical activity of the brain, the electroencephalogram, or EEG, signal is typically employed for its diagnosis. The interpretation of EEG signals can be used to improve more advanced forms of human interaction [11,13,14]. EEG recordings are now done digitally so that they may be seen on a computer display device. This also makes it possible for the recordings to be automatically evaluated. The use of standardized terminology for seizure categorization also makes it simpler for clinicians who care for people who have epilepsy and who conduct research on epilepsy to communicate with one another. Since the regular functioning of many brain cells is disrupted during an epileptic seizure, the electroencephalogram can be used to detect the onset of a seizure (EEG). Because effective seizure management is contingent on making an accurate diagnosis, it is critical to determine whether or not a patient suffers from epilepsy, as well as the specific type of disorder they have. The categorization of seizures into their various subtypes is helpful for guiding subsequent testing, treatment, and assessment of prognosis or outlook. During an EEG recording, the most difficult part is trying to locate the ictal spikes and seizures. In order to detect epileptic activity, it is necessary for a specialist to perform an in-depth analysis of the entire duration of the EEG recordings [15], which can be a time-consuming process. Both the sheer volume of long-term EEG recordings and their growing prevalence in clinical practice have contributed to a reduction in the likelihood that a specialist will incorrectly interpret the data or fail to arrive at the correct detection.

Different researchers have created a DL model to effectively detect epileptic from EEG signals so as to address the problem described in the previous paragraph. Various machine learning and deep learning (ML/DL) models have been used in previous publications [2,16] to help discover important and distinguishable features associated with biomedical imaging and signal for use in the binary classification task [2,16,17,18,19,20,21,22,23,24,25]. Deep learning has been responsible for a significant amount of the success that has been seen in the automated feature extraction and categorization of EEG epileptic seizures [6,10,15,17,18,19,20,21,22,23,24,25,26]. Despite this, there is still space for development in this area. According to the existing studies, the proper detection of epileptic seizures using EEG signal analysis utilizing hybrid deep learning models is always appropriate, proving the efficacy and efficiency of the technique [2,15,17,19]. This is the case irrespective of the condition of the literature. As a consequence of this, the objective of the hybrid deep learning model that is proposed in this paper is to improve the performance of previously developed EEG seizure classifiers. The following is a list of the most important findings from the study:

- A hybrid of recurrent neural networks (RNNs) and bi-directional long short-term memories (BiLSTM) is proposed for the purpose of automatically identifying epileptic seizures through the processing of EEG signals.
- The efficacy of the newly developed model is validated by conducting a complete comparison to the existing state-of-the-art learning models.
- The recommended method provides a number of advantages, including shorter periods of time needed for detection, a reduced proportion of false positive results, increased sensitivity, and increased specificity.

The paper is being organized as follows: The state-of-the-art of seizure detection is outlined in Section 2. In Section 3, we discuss the data and our hybrid RNN-BiLSTM model. In Section 4, the findings of the experiments are presented, along with a discussion of the study’s conclusions and directions for further research.

## 2. Literature Review

In the last decade, numerous methods for feature extraction and classification approaches have been developed in order to improve the overall performance of the constructed classifier and expand its capacity for seizure detection. Both the continuous wavelet transform (CWT) and the discrete wavelet transform (DWT) are examples of traditional machine learning techniques used to extract features for the goal of building a classifier for the identification of epileptic seizures [27,28,29,30]. The adequacy of the detection of seizures has been strengthened by the help of DL-based techniques [6,10,15,17,18,19,20,21,22,23,24,25,26]. The introduced algorithms aim at boosting diagnostic standards and automating identifying essential features. To better categorize epileptic seizures, Choi et al. [21] presented a hybrid model that combines one-dimensional convolutional networks (1D CNN), gated recurrent unit networks (GRU). The introduced model has an overall accuracy of 82.86 percent, along with a sensitivity of 80% and a precision of 85 percent. A gated recurrent network, also known as GRN, was described by Affes et al. [31] as a method for predicting epileptic seizures using features collected from EEG data. These features reflect the temporal aspect and the frequency aspect of the signal, respectively. Using data that were collected in the Children’s Hospital of Boston, GRN was able to predict epileptic seizures with an accuracy of 75.6% and a mean sensitivity of 89%. A DL-based framework has been presented by Raghu et al. [32] for the categorization of seven kinds of seizures alongside non-seizure EEG. This was accomplished by the utilization of convolutional neural networks and transfer learning. The model was trained and evaluated using the EEG signals collected from Temple University Hospital. The approaches of transfer learning and feature extraction, taken separately, led to the greatest possible classification accuracy of 88.30% when using Inceptionv3 pretrained DNN. The Deep Clinical Sparse Autoencoder, also known as the DCSAE, was applied by Hilal [6] and colleagues in order to classify epileptic seizures based on EEG signals collected from the benchmarking UCI-Epileptic dataset. In order to extract the important features, the coyote optimization technique has been implemented; after that, the selected features are input into the DCSAE model. They have achieved a specificity of 99.2%, sensitivity of 99.19%, and accuracy of 98.67%. The dataset, UCI-Epileptic, has been addressed by Mursalin et al. [33]. They presented an innovative method for identifying the important elements from a benchmarking EEG data taken from the University of Bonn. The collected features have been put through a number of different traditional classifiers, such as the SVM, Random Forest (RF), and the k-nearest neighbor (KNN). By utilizing an RF classifier, they were able to attain a success rate of 98.7%. A multi-view convolutional neural network, or CNN, framework was presented by Liu et al. [10] for the purpose of predicting the occurrence of epilepsy seizures. The researchers’ objective was to acquire significant features in the time/frequency domain. They were able to get an average area under the curve of 0.82 and 0.89 on two participants of the CHB-MIT dataset. In order to improve the effectiveness of traditional machine learning approaches (such as Support Vector Machines and Neural Networks) in EEG classification, Nagabushanam et al. [23] developed a method that combines the long short-term memory (LSTM) with the neural network (NN). The level of accuracy that was reached was 71.38%. The independently recurrent neural network, a new deep learning model, was recently developed by Yao et al. [34] to build a convenient method for the seizure/non-seizure classification. The suggested method may extract temporal and spatial information from the short-term to long-term range of the complete record. Cross-subject experiments were used to evaluate the CHB-MIT data, which is notoriously noisy. They accomplished 87% accuracy, 87.3% sensitivity, and 86.7% specificity.

A hybrid RNN and LSTM was proposed by Najafi et al. for the purpose of diagnosing epilepsy in [2]. After the RNN was deployed for the feature selection process, the long short-term memory was put to use for the epilepsy classification stage. At the outset, both normal and epileptic LB channels were broken down into three levels, and 15 distinct characteristics were gleaned from each level. The LSTM was then given the selected features to use in its classification process after they had been retrieved from each signal segment individually. The investigation’s findings showed that the proposed algorithm was successful in differentiating normal participants from epileptic subjects with a rate of 96.1% accuracy, 96.8% sensitivity, and 97.4% specificity. Because of the excellence of the RNN-LSTM technique in extracting significant spatial and temporal features from EEG signals, as well as its superior accuracy and sensitivity in epilepsy classification, we were motivated to investigate other similar fusion models based on recurrent neural networks and the bidirectional LSTM. This was made possible by the fact that the RNN-LSTM technique is effective in extracting significant spatial and temporal features from EEG signals. Bidirectional long short-term memory, or BiLSTM for short, is a type of recurrent neural network that is most commonly utilized in natural language processing. In contrast to a conventional LSTM, the input travels in both directions, and the system is able to make use of information from both sides. In addition to this, it is an effective method for modeling the sequential dependencies that exist between words and phrases in both the forward and backward directions of the sequence. As a consequence of this, an RNN-BiLSTM model was presented in this paper for the automated extraction of attributes from EEG data. The purpose of this model is to improve the classification’ accuracies, and sensitivities of these signals. In addition, the combination of bidirectional LSTM and RNNs model has demonstrated superior performance in the classification/forecasting tasks of other non-medical applications [35,36]. Because of this, it has been implemented and evaluated with the intention of improving the existing performance of the state-of-the-art regarding the classification of elliptic seizures.

## 3. Materials and Methods

**Figure 1:**
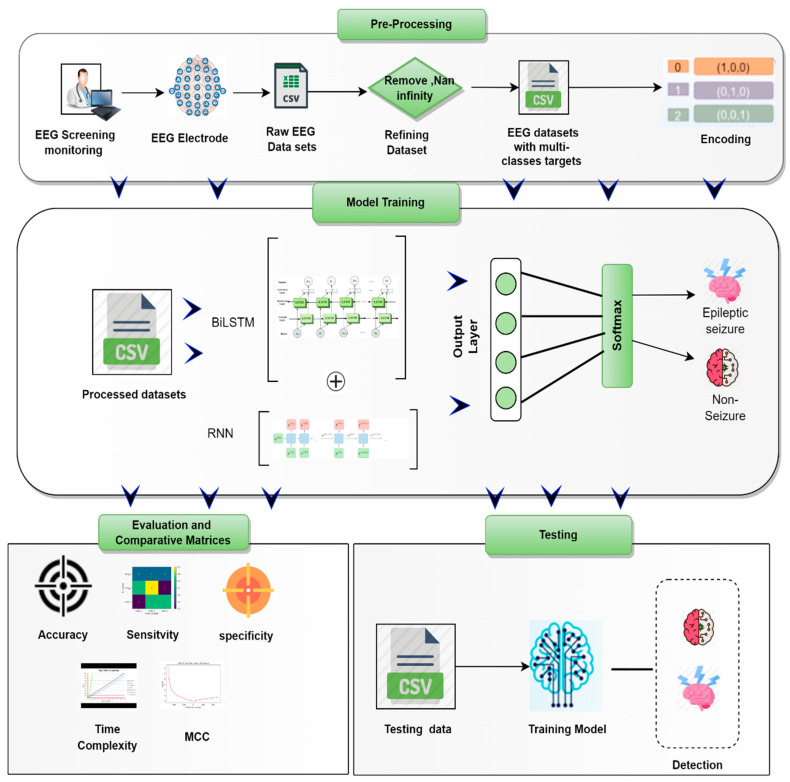
Proposed RNN-BiLSTM model for detecting epileptic seizures.

Important procedures for applying the suggested model (RNN-BiLSTM) to detect epileptic episodes are outlined below. The process of seizure detection normally consists of two phases. It begins with gathering and cleaning data, then moves on to the suggested model’s automatic feature extraction and selection. This study’s primary structure is shown in Figure 1. The proposed framework’s primary components are the following steps: EEG signal preprocessing; model training; model testing; and model evaluation.

### 3.1. Data Collection and Preprocessing

**Figure 2:**
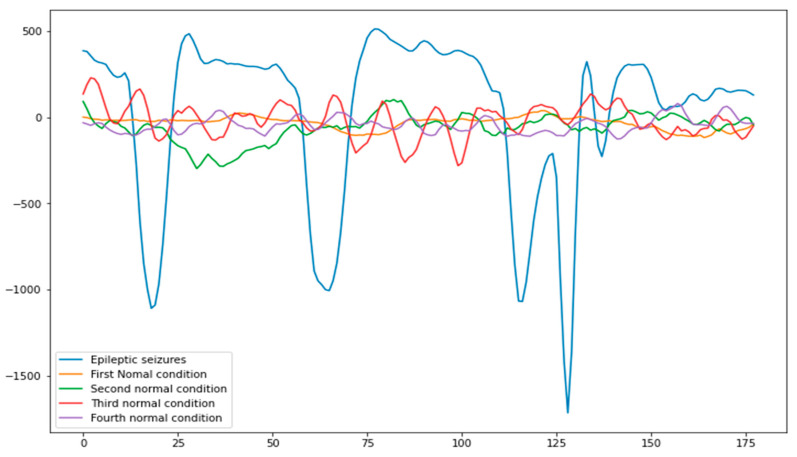
Data classes with frequency dimensional values.

This study was conducted using EEG data collected at the University of Bonn as a benchmarking dataset [37] and is split into five different directories and a hundred files per folder, each of which represents a particular sample or person. Each file stores the mental activity of the subject for 23 min, and 6 s. 4097 discrete data points representing the appropriate time series have been gathered. Each data point illustrates a different sampling rate of the electroencephalogram (EEG). Therefore, there are 500 individuals, and for each individual there are 4097 data points spanning 23.5 s. Data from each of the 4097 samples were broken up into 23 sets, each of which is 1 s long and contains 178 individual data points. Therefore, we have 11,500 rows of data (rows), each of which has 178 points of data of 1, and the last column is labeled y. Since each data point reflects the value of an EEG recording at a particular point in time, the chunks were randomly shuffled after division (1,2,3,4,5). In column 179, the variable representing the outcome is indicated by the letter y, while the variables representing the predictors are shown by the letters X1, X2, etc.… X178. The value that is recorded in the variable y represents the subcategory of the 178-dimensional input signal. More specifically, y values that are within the range [1, 2, 3, 4, 5]. In column 179, the outcome variable is represented by the letter y, and the predictors are indicated by the letters X1, X2, … X178, as shown in Figure 2. The y variable stores the 178th dimension of the input signal’s granularity. In this case, y is within the interval [1, 2, 3, 4, 5]. Having the patient’s eyes open during the EEG recording time indicates a value of y = 5. This means the patient was awake. The patient’s eyes were closed during the EEG recording if y = 4, indicating the patient was feeling sleepy. In addition, if y = 3, it means that the tumor has invaded the EEG-normal region of the brain. Moreover, if y = 2, then the EEG was taken from the area where the tumor was located. Moreover, if y equals 1, then seizure-related activity will be recorded. In this research, we used a binary classification approach to the identification of epileptic seizures, where a label of 0 indicates the of an epileptic seizure and labels of 1, while 2, 3, 4, and 5 indicate the absence of an epileptic one. In this experiment we used binary classification epileptic seizure versus non-epileptic seizure detection, so the class label from 2 to 5 was assigned 0, which represents the normal condition of EEG epileptic seizure detection, while 1 represents the patients have epileptic seizures, as shown in Table 1.

### 3.2. The Proposed RNN-BiLSTM Learning Model

Recurrent neural networks (RNNs) are a type of artificial neural network in which the effects of some nodes’ output on other nodes’ subsequent input can be cycled through. Because of this, it is able to display temporal dynamics in its behavior. Recurrent neural networks (RNNs), which are a descendant of feedforward NNs, have the ability to memories internal states to process sequences of inputs of varying lengths [38,39,40,41]. In an RNN, all of the input vector components share nearly identical weights, as opposed to the feedforward NNs where each component has its own weight. In most cases, recurrent networks outperform traditional methods because they combine the weights of multiple input vector positions into a single vector. Sequences of varying lengths can be processed by the same model by simply reusing the weights. This reduces the number of network-learning parameters (weights), which is an additional benefit. Furthermore, the outputs passed on to the next phase were both calculated using the input vector and several data from the preceding step (usually another vector). Units are shorthand for the formulas used to derive the intermediate results (blocks). Therefore, for the most basic form of a recurrent network, the relation that follows, Equations (1) and (2), can be used to define a block:

(1) $$o^{\langle t\rangle }=f_{1}(W_{oo}o^{\langle t-1\rangle }+W_{ox}x^{\langle t\rangle }+b_{o})$$

(2) $${\hat{y}}^{\langle t\rangle }=f_{2}(W_{yo}o^{\langle t\rangle }+b_{y})$$

where $x^{\langle t\rangle }$ is an input sequence vector, and *t* denotes the iteration at which recurrent relations are computed. The $f_{1}$, $f_{2}$ represent the activation functions. The weight matrices and biases are denoted by the symbols $W_{oo}$, $W_{ox}$, $W_{yo}$, $b_{o}$, and $b_{y}$, respectively.

In the realm of artificial intelligence, LSTM [24,41,42,43,44] is a subset of recurrent neural networks. The disappearing of gradients problem is addressed by the introduction of this architecture. Further, this network type is superior at keeping long-range connections alive by understanding how values at the beginning and end of a sequence are related. In the LSTM model, these expressions take the form of gates. There are three distinct varieties of gates: The forget gate regulates how much data are sent from one memory cell to another. The memory cell’s update-gate (or update input) is the gate responsible for determining if the cell will be updated. Additionally, it regulates the amount of data that a possible new memory cell can send to the current memory cell. The next hidden state’s value is determined by the output gate. 

In this study, we introduce the fusion of the RNN and BiLSTM for binary class epilepsy identification. The network consisted of five layers: a sequence input layer, an RNN layer with 100 hidden units, a bidirectional LSTM layer with 200 hidden units, an FC layer, a SoftMax layer, and a classification output layer. The user-specified values for the deep learning layers are shown in Table 2, along with brief descriptions of their function. 

**Figure 3:**
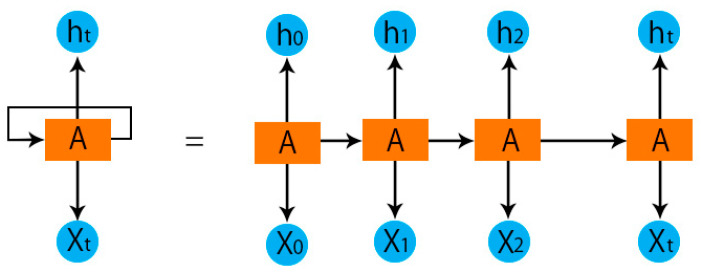
RNN schematic.

The RNN module is based on the schematic depicted in Figure 3. A specific type of RNN structure, the binary long short-term memory (LSTM) network, has been shown to be reliable and effective for simulating a sequence that may be used for various purposes and has lengthy dependencies in research that include time [45].

**Figure 4:**
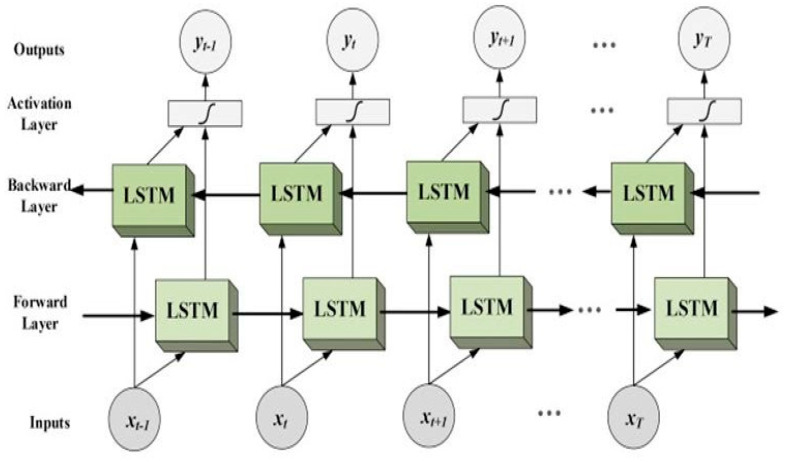
BiLSTM schematic.

After the RNN module, a BiLSTM layer was used. Since the collected EEG signals are arranged in a time-sequence based fashion, the present state is strongly influenced by the past environments. To address this problem, the BiLSTM model is the most efficient tool at one’s disposal. Several self-parameterized regulating gates in the BiLSTM module’s memory cell allow for the reading, writing, and clearing of state information, as shown in Figure 4. If that door were to be opened, the cell’s entire informational capability would become available. To recap, the previous cell’s process failed because the forget gate was not turned on. Assuming nothing takes place, the preceding data may be “forgotten.” As can be seen in Figure 4, the output gate can decide for itself if it needs to relay the most recent cell output and the ultimate state.

In order to address the problem of overfitting, we made use of two dropout layers that were positioned below the ReLU activation function, in addition to the BiLSTM layer. Using these dropout layers allows us to avoid the problem of overfitting. In the meantime, the dropout contributes to the objective of minimizing the error of generalization, which is being pursued in conjunction with the growth in the number of layers contained within neural networks. After the BiLSTM layer comes the FC layer, then the SoftMax layer, and finally the classification layer.

### 3.3. Model Training and Testing

The use of cross-validation is a reliable strategy for avoiding overfitting. The entire dataset has been divided up into sections. In order to perform a typical K-fold cross-validation, the data need to be divided up into k folds. Then, we used the remaining holdout fold as the test set while iteratively training the algorithm on k-1 folds.

In this research we used k cross-validation on to 5-fold. We used the four given folders to train the models, then tested the models on the last folder. This process repeated five times to produce a reliable and practical detection system of epilepsies from EEG signals. Determining the learning parameters for training the DNN that would yield the optimum performance is a complex optimization issue due to the large complexity and non-convexity of the objective function. In this case, the standard practice is to employ stochastic optimizer. This research makes use of the ADAM optimizer with the following settings: learning rate = 0.0001, batch size of 32, and a number of epochs of 400. These parameters were chosen by analyzing experimental validation findings and were applied uniformly across all networks for fair evaluation of their performance and resource requirements.

## 4. Results and Discussion

In order to carry out experiments, we made use of both a graphics processing unit (GPU) and a central processing unit (CPU) manufactured by Intel and bearing the model number Core i5-7700. In addition, the suggested procedures were trained with the assistance of Keras and Python version 3.6. Accuracy (Acc), Precision (Prec), Sensitivity (Sen), and Specificity (Speci) are the four-performance metrics that we used to evaluate the proposed model. While accuracy is the proportion of a sample over the entire population that has been correctly classified, sensitivity is the percentage of true positives that have been accurately detected or correctly identifies patients as having epileptic seizures. Sensitivity is more accurately referred to as the true positive rate (TPR), or Recall. Accuracy is presented as a sample over the entire population that has been correctly classified. Specificity demonstrates that the patents in question do not involve any epileptic seizures. Specificity can be referred to as the true negative rate (TNR). The mathematical equations for all of the important performance metrics are listed below, in Equations (2)–(6). The TPost, TNeg, FPost, and FNeg are the true positive, true negative, false positive, and false negative, respectively. The TNeg output indicates the number of correctly classified negative examples. A similar notation, TPost for true positive, denotes the percentage of correctly identified positive examples. A false positive value FPost indicates the number of falsely positive examples that were incorrectly labeled as negative (FNeg), which indicates the opposite number of falsely negative examples that were incorrectly labeled as positive.

(3) $$Acc\left(\%\right)=\frac{TPost+TNeg}{TPost+TNeg+FPost+FNeg}\;$$

(4) $$Prec\left(\%\right)=\frac{TPos}{TPos+FPos}$$

(5) $$TPR=Sen\left(\%\right)=\frac{TPos}{TPos+FNeg}\;$$

(6) $$TNR=Speci\left(\%\right)=\frac{TNeg}{TNeg+FPos}$$

In addition to these metrics, a number of other metrics, such as the Confusion matrix, Matthew’s correlation coefficient, the Roc Curve, false omission rate (FOR), false discovery rate (FDR), and Negative Predictive Value, were used to perform an analysis on the results. The confusion matrix is a specialized form of the contingency table. Contingency tables summarize binary decision-making. It has two dimensions, which are labeled actual and predicted, and both dimensions contain identical sets of classes [46]. Dealing with an imbalanced target variable is a common challenge that arises when applying machine learning techniques to medical applications. When machine learning techniques are used on data that are not evenly distributed, using confusion matrices as error measures is insufficient. Therefore, in this work, we make use of the Matthews correlation coefficient, also known as MCC, which is recognized as an important error measure for data that are not balanced [47,48]. The Matthews correlation coefficient, also known as r, is a statistical measure that compares the true classes, also known as TC, to the predicted labels, also known as PL [49]. Its definition can be found in Equation (7), as shown.

(7) $$MCC=\frac{cov\left(TC,PL\right)}{\sigma_{TC}\times \sigma_{PL}}=\frac{\left(TPos\times TNeg\right)-\left(FPos\times FNeg\right)}{\sqrt{\left(TPos+FPos\right)\left(TPos+FNeg\right)\left(TNeg+FNeg\right)\left(TNeg+FNeg\right)}}$$

where $\sigma_{TC}$, and $\sigma_{PL}$ are the variances of the true classes TC and the predicted labels PL, and cov(TC,PL) is the covariance between them. The MCC can range anywhere from −1 to +1. When the value is −1, it indicates that the prediction is completely inaccurate, whereas when the value is +1, it indicates that the prediction is spot on. When MCC is equal to zero, it indicates that we are thinking about a random classification in which the model predictions do not have any discernible correlation to the actual results. 

The area under receiver operator characteristic, ROC curve, is a different type of error metric that we are presenting. The TPR, or sensitivity, is displayed on the ROC curve in relation to the FPR, or specificity [50]. The false discovery rate and the false omission rate are two additional error metrics we have employed [51]. Biology and medicine are common application domains for the FDR and the FOR [52,53]. FDR calculates the percentage of erroneous findings among a group of significant hypothesis tests. The complement of the negative predictive value, false omission rate, is a statistical technique used in multiple hypothesis testing scenarios to account for numerous comparisons. It calculates the percentage of false negatives that are wrongfully rejected. Equations (8) and (9) show the definitions of FDR and FOR. Both measures have a range of 0 to 1. For an errorfree classification, we have FNeg=FPos=0, implying FDR=FOR=0. In contrast, when TPos=TNeg=0, we get FDR=FOR=1.

(8) $$FDR=\frac{FPos}{FPos+TPos}$$

(9) $$FOR=\frac{FNeg}{FNeg+TNeg}$$

The negative predictive value is another error metric for evaluating classification accuracy in the medical domain (NPV) [54]. Given a negative test result, NPV is the likelihood that a person does not have a disease or condition. The percentage of people with negative test results who are correctly identified or diagnosed is what NPV stands for. In other words, when a person receives a negative, normal, test result, NPV represents the likelihood that the person is healthy (e.g., intellectually normal). The definition of NPV is given in Equation (10).

(10) $$FOR=\frac{TNeg}{TNeg+FNeg}\;$$

**Figure 5:**
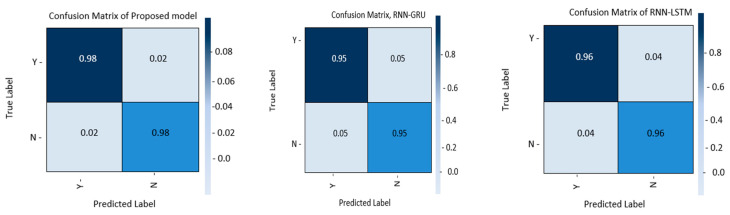
RNN-LSTM, RNN-GRU, and the proposed RNN-BiLSTM model’s confusion matrices.

We have evaluated the performance of the hybrid model that has been proposed, known as RNN-BiLSTM, as well as other models (RNN-LSTM and RNN-GRU) that are comparable to it, that are currently available in the state-of-the-art for the identification of epileptic seizures. Figure 5 presents the confusion matrices that were calculated for the three different models. The combination of RNN and BiLSTM is shown to correctly recognize the classes after being subjected to an in-depth experiment involving the confusion matrix. The proposed model for the classification of epileptic seizures achieves the TPost, TNeg percentages of 0.98 and 0.98, respectively. On the other hand, the values that were achieved for the same measures by using the RNN-LSTM and the RNN-GRU were respectively 0.96 and 0.95 and 0.97 and 0.97. In conclusion, the method RNN-BiLSTM was superior to the other two methods, RNN-LSTM and RNN-GRU, in terms of its accuracy in detecting the classes as well as its performance.

**Figure 6:**
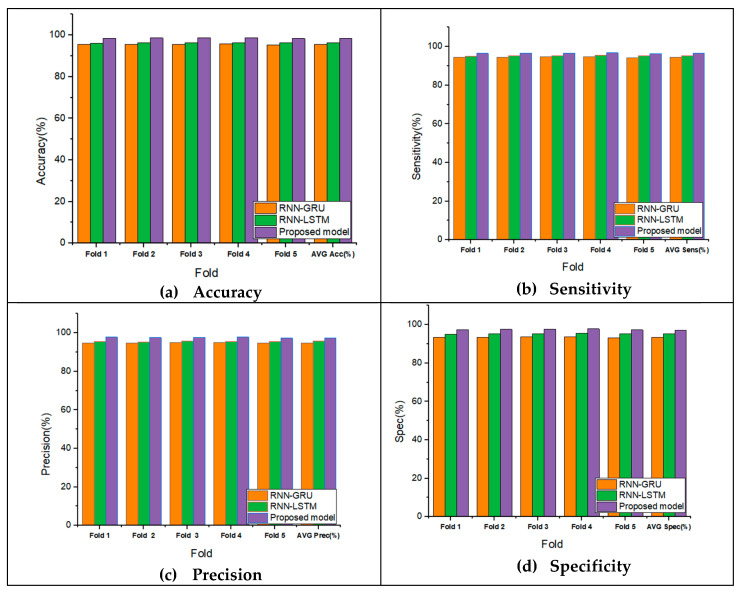
The attained performance measures of proposed models and RNN-LSTM, RNN-GRU models on various folds (1–4).

In the experimental study, the performance measures on different folds from 1 to 5 are shown in Figure 6. The average accuracy, sensitivity, precision, and specificity have each been improved using the RNN-BiLSTM that we have proposed, which has achieved average values of 98.4%, 98.50%, and 97.40%, and 97.30%, respectively. The results demonstrate that the new model achieves improvements in the same metrics that are average performance measure 2.1%, 2.69%, 1.95%, and 2.2%, respectively, compared to RNN-LSTM and RNN-GRU models.

**Figure 7:**
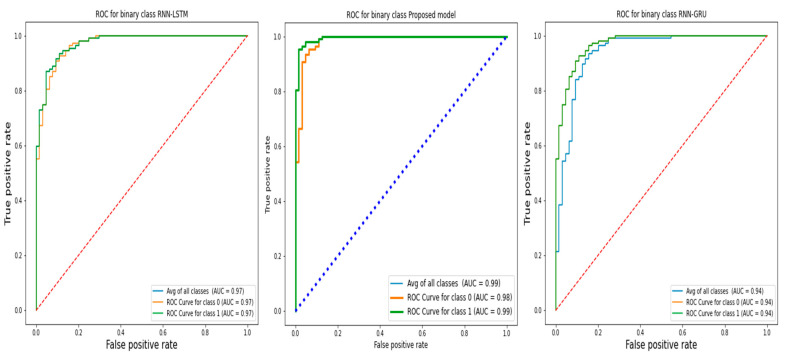
The ROC of the proposed models and RNN-LSTM, RNN-GRU model on 5 folders.

The ROC is regarded as an essential metric in all EEG epileptic seizure detection. The ROC plots the results of the comparison between the true negative and true positive rates. Figure 7 depicts the ROC of the suggested approaches, which clearly depict the link between true positives and true negatives. The suggested model outperforms the RNN-LSTM and RNN-GRU in terms of average ROC values.

**Figure 8:**
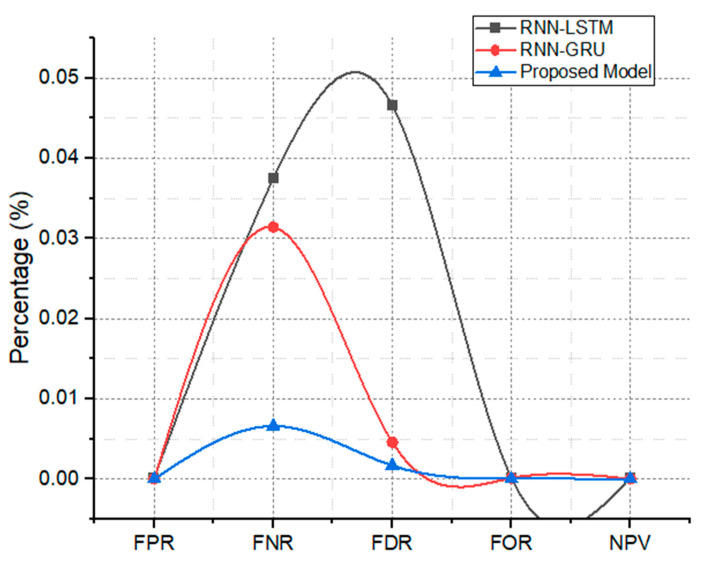
FDR, FOR, NPV, FNR, and FPR analysis of proposed model and RNN-LSTM, RNN-GRU models.

The sensitivity and specificity attained by the proposed models has been compared to its attained false discoveries (FDR, FOR, and NPV), as shown in Figure 8. The figure also depicts the achieved results for the same metrics for the state-of-the-art models (RNN-LSTM, RNN-GRU model.). As can be seen in the figure, the suggested model achieves high sensitivity and specificity while simultaneously producing low false discoveries (FDR and FOR). 

As a measure of the correlation between the correct and incorrect labels, the Matthews correlation coefficient was computed for the proposed models and RNN-LSTM, RNN-GRU models. The value for the MCC that measures the correlation between the true classes, TC, and the predicted labels using the proposed model has been recorded as 0.99. On the other hand, the attained values for the MCC are 0.90 and 0.89 for the RNN-LSTM, RNN-GRU models. MCC measures the correlation between the correct and incorrect labels. When the classifier is perfect (FPos=FNeg ≅ 0) the value of MCC is almost 1, indicating perfect positive correlation. In this, the proposed model indicated 0.99, which is close to 1, meaning that both classes are predicted well compared to other models.

**Figure 9:**
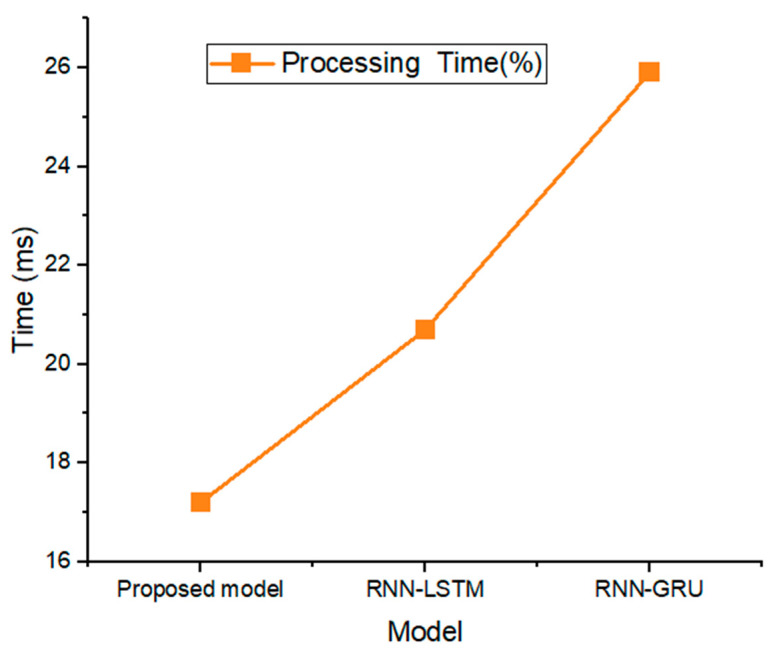
Processing time (%) of proposed model and RNN-LSTM, RNN-GRU models.

Figure 9 depicts the processing time for the suggested model. We have estimated the testing time for the suggested model to be equal to the detection time. Since much of the work during the training phase is done away from the main system, it is ignored. However, it is generally agreed that testing is crucial because it proves the model works as intended. With a processing time of only 17.1 (MS), our proposed method is clearly computationally efficient. RNN-LSTM also needs less time for testing than RNN-GRU does.

## 5. Comparing the Proposed Model to Traditional ML Models

The proposed model’s performance has been analyzed further by comparing it to those of the most conventional ML models like KNN, SVM, and RF [55]. Transfer learning has been used to extract significant features for use in traditional classification techniques. To begin, the high-level deep features were extracted using a pretrained convolutional neural network (CNN) named AlexNet in order to bypass the overfitting problem through the transfer learning technique [56,57,58]. Once the deep features are acquired, they are given to the standard classifier mentioned above. As a part of the feature extraction process, we used AlexNet that had already been trained. Data of the image kind were fed into the AlexNet at the input layer. Therefore, in this study, we saved each EEG as a separate image. The input images have been resized into 227 × 227, the standard size for the AlexNet network.

Model parameters are used to define or express a model in ML/DL. The training phase, on the other hand, necessitates picking the right hyperparameters for the learning algorithm to utilize in order to discover the right mapping between the features and the targets in order to reach intelligence [59]. Parameters known as hyperparameters are used to regulate and set the values of model parameters that a learning algorithm ultimately learns. The learning parameters of AlexNet were tested during its training. Here, we employed a stochastic gradient descent with momentum (SGDM) optimizer. With this optimizer, we used a learning rate of 0.001, mini batches of 32, and 400 training epochs. The validation findings from the ongoing experiments were used to choose these hyperparameters.

Different hyperparameter values were tested throughout this study; the values that yielded the best results during training for the conventional SVM, KNN, and RF classification models are detailed in Table 3. The random forest classification technique makes use of many trees, and we have the ability to determine the minimum and maximum values for the number of trees required by the algorithm. This is accomplished with the use of a hyperparameter called “n estimators”. In this research, the optimal classification performance could be obtained by setting this hyperparameter to a value of 10 trees. It is well-known that KNN is an effective supervised classification algorithm. The similarity between the input features and the target is used by KNN to estimate the relationship between them. KNN is a classification method that uses the opinion of the input instance’s most similar neighbors (a number denoted by “k”) to determine the correct class. To determine the best value of k, we tested the KNN algorithm on the training dataset with several values of k and found that k = 5 yielded the best results. SVM seeks a hyperplane in the input feature space to clearly categorize the input data points in a classification task. Selecting the plane that maximizes the gap between the distributions of two classes of data is the optimization issue posed by finding the hyperplane. In this research, we have tried out a variety of kernel functions for use in SVM training. For EEG data classification, the sigmoid kernel function has proven to be the most effective.

**Figure 10:**
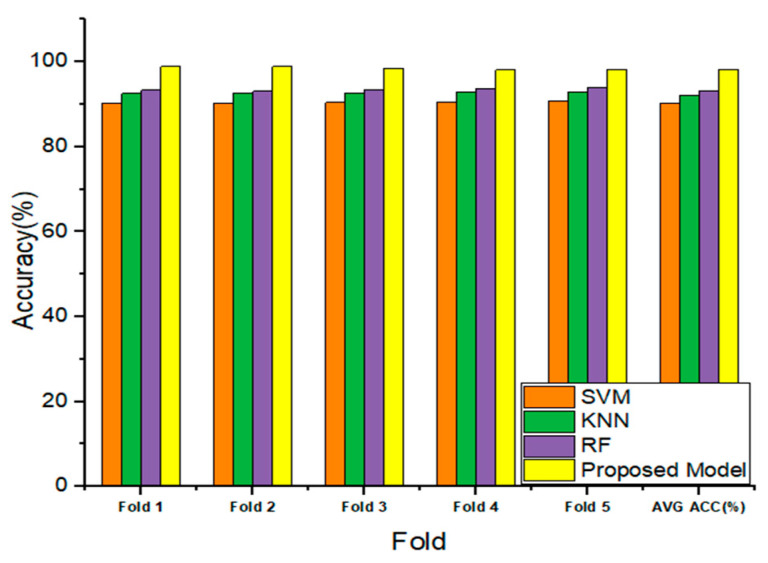
Comparing the attained accuracy of the proposed model to the conventional ML techniques (KNN, SVM, and RF) on 5-folds cross validation.

The findings were obtained by the application of a cross-validation procedure that was carried out five times. The cross-validation test is illustrated by a bar chart in Figure 10, which compares the retrieved accuracy of the proposed model to that of the classical classifier in each fold. As depicted in Figure 10, the proposed model has outperformed the classical classification methods in the classification of epileptic/non-epileptic seizures.

## 6. Comparing the Performance of the Proposed Model to State-of-the-Art

In this part of the article, we have additionally compared the model against a number of benchmark methods that currently exist. The findings of the evaluation in relation to the most recent benchmarks are presented in Table 3. In terms of the evaluation metrics known as Acc, Pres, Sens, and Spec, the proposed techniques, known as RNN-BiLSTM, offer superior results. Table 4 shows that the created hybrid model outperforms previous efforts by Yao et al. [34] and Raghu et al. [32]. The performance boost achieved by hybrid RNN-LSTM [2] has been further enhanced by the suggested hybrid RNN-BiLSTM. It has also produced high performance, comparable to that of work done with the Deep Clinical Sparse Autoencoder. The suggested hybrid RNN-BiLSTM framework system has achieved comparable performance to that of Hilal et al. [6] and Mursalin et al. [33] on the benchmarking UCI-Epileptic dataset. Hilal et al. [6] employed the Deep Clinical Sparse Autoencoder for the classification of epilepsy UCI-Epileptic dataset, and Mursalin et al. [33] have analyzed the UCI-Epileptic dataset using hybrid metaheuristic feature selection and traditional ML-based classifiers. Table 4 shows that the suggested system can achieve comparable performance to the other systems compared.

## 7. Conclusions

Through an analysis of the patient’s EEG signal data, the RNN-BiLSTM model is presented as a method for identifying epileptic seizures that is found in this study. The RNN is extremely effective at extracting features from EEG signals, whereas the BiLSTM network is able to classify the collected data. This research incorporated publicly accessible EEG data from UCI Machine Learning repository for epileptic seizure classification, and the results serve as evidence that the proposed method is accurate in predicting clinical outcomes. The epileptic seizure recognition process consists of a total of one exam, which also includes binary recognition tasks. The accuracy, precision, sensitivity, and specificity of the proposed system have been thoroughly evaluated using a robust collection of error measures, such as the confusion matrix, the Matthews correlation coefficient, the Roc Curve, the false omission rate, the false discovery rate, and the negative predictive value. Our proposed RNN-BiLSTM has increased accuracy, sensitivity, precision, and specificity to 99.4%, 98.99%, 99.05%, and 98.8%, respectively. The data show that the new model improves the same measures by 1.8%, 1.69%, 1.95%, and 2.2%. The proposed approach reaches high levels of sensitivity and specificity while also generating a minimal number of false discoveries (FDR and FOR). In addition, the proposed method was judged against approaches that are now considered to be state-of-the-art, and it was found to be a more accurate classification of such methods.

Although the proposed method has made significant headway in the field of epileptic seizure detection, there are still some issues that need to be handled in a more comprehensive manner in the near future. When applied to multi-class issues, the strategy that has been provided does not have a recognition accuracy that is particularly extraordinary or even good. Second, in order to carry out supervised training with the suggested approach, a sizeable amount of EEG signal data that have been labeled will be required.

## Figures and Tables

**Table 1:** Explanation of the UCI-epileptic seizure dataset.

Class Description	No. of Samples	Class Labels	Binary Classification Samples
seizure	2300	1	2300
First normal (Before seizure the signal of the patient)-	2300	0	9200
Second normal (Healthy brain EEG recorded data)-	2300	-	-
Third normal—Eyes closed have no seizure	2300	-	-
Fourth normal—Eyes opened have no-seizure	2300	-	-

**Table 2:** Layers in the proposed hybrid RNN-BiLSTM model.

Layers	Other Parameters	Value
RNN layer	Batch size = 100 Epochs = 100 Learning rate = 0.01 ADAM	Hidden 100 Units
BiLSTM		Hidden 200 Units
FC layer		2 FC layers-
SoftMax		Cross entropy
Classification Layer		

**Table 3:** Hyper-parameters for the training of the conventional classifiers, including KNN, SVM, and RF.

Classifier	Learning Parameters
SVM	Kernel function = Sigmoid Kernel Function
KNN	K = 5
RF	n-estimators = 10

**Table 4:** Comparing the performance of the introduced model, RNN-BiLSTM, to state-of-the-art epileptic seizures detection systems.

Publication	Method	EEG Class	Dataset	Acc (%)	Sens (%)	Spec (%)
Yao et al. [34]	Independent RNN	binary	CHB-MIT	87%	87.3%	86.7%
Raghu et al. [32]	Transfer learning and CNN	8 classes	Temple University Hospital EEG signals	88.3%	-	-
Choi et al. [21]	hybrid model (1D CNN and GRU)	binary	Asan Medical Center Children’s Hospital	82.86 %	80%	-
Najafi et al. [2].	hybrid RNN and LSTM	binary	HCTM hospital’s EEG data	96.1%	96.8%	97.4%
Hilal et al. [6]	Deep Clinical Sparse Autoencoder	binary	UCI-Epileptic	98.67%.	99.19%	99.2%
Mursalin et al. [33]	Hybrid metaheuristic Feature selection, and traditional ML-based classifiers	binary	UCI-Epileptic	98.7%	-	-
**Proposed model**	**Hybrid RNN-BiLSTM model**	**binary**	**UCI-Epileptic**	**98.4%**	**98.30%**	**98.10%**

## Data Availability

Not applicable.
